# Detection of multivessel calcific disease progression in a patient with chronic limb-threatening ischemia using fluorine-18 sodium fluoride positron emission tomography imaging

**DOI:** 10.1016/j.jvscit.2023.101137

**Published:** 2023-03-04

**Authors:** Ting-Heng Chou, Molly K. Wynveen, Eleanor T. Rimmerman, Surina Patel, Michael R. Go, Mitchel R. Stacy

**Affiliations:** a Center for Regenerative Medicine, The Abigail Wexner Research Institute, Nationwide Children's Hospital, Columbus, OH; b Interdisciplinary Biophysics Graduate Program, The Ohio State University College of Medicine, Columbus, OH; c Division of Vascular Diseases and Surgery, Department of Surgery, The Ohio State University College of Medicine, Columbus, OH

**Keywords:** Calcification, Chronic limb-threatening ischemia, Diabetes mellitus, Positron emission tomography, Sodium fluoride

## Abstract

Vascular calcification contributes to morbidity and poor clinical outcomes for patients with peripheral artery disease; however, the traditional assessment of the calcium burden using computed tomography (CT) imaging or angiography represents already established disease. In the present report, we describe a 69-year-old man with chronic limb-threatening ischemia who had undergone positron emission tomography/CT imaging with fluorine-18 sodium fluoride to evaluate the relationship between baseline levels of positron emission tomography-detectable active vascular microcalcification and CT-detectable calcium progression 1.5 years later. CT imaging at follow-up identified progression of existing lesions and the formation of new calcium in multiple arteries that had demonstrated elevated fluorine-18 sodium fluoride uptake 1.5 years earlier.

High levels of arterial calcification above and below the knee can reduce limb blood flow and promote deficits in muscle perfusion, limit options for revascularization, increase the incidence of ulceration and nonhealing wounds, and contribute to worse revascularization outcomes for patients with peripheral artery disease (PAD).^1^, ^2^, ^3^, ^4^ Thus, a need exists for an imaging strategy that can detect the early active stages of calcific disease in the lower extremities to improve noninvasive monitoring and the detection of patients at risk of calcific disease progression.

Positron emission tomography (PET)/computed tomography (CT) imaging studies using the radionuclide fluorine-18 (^18^F)-sodium fluoride (NaF) have demonstrated the potential of this imaging strategy for quantifying active stages of microcalcification in the cardiovascular system.^5^^,^^6^ However, to date, application of ^18^F-NaF PET/CT imaging has not been used in the vascular medicine community as a tool for monitoring and predicting disease progression in patients with PAD. In the present report, we describe the case of a man with chronic limb-threatening ischemia (CLTI) who had undergone baseline ^18^F-NaF PET/CT imaging and follow-up CT imaging studies 1.5 years later to evaluate the relationship between the arterial uptake of ^18^F-NaF and subsequent calcific disease progression in each primary artery of his symptomatic limb. The institutional review board and radiation safety committee of Nationwide Children's Hospital approved the study protocol. The patient provided written informed consent for the report of his case details and imaging studies.

## Case report

**Fig 1:**
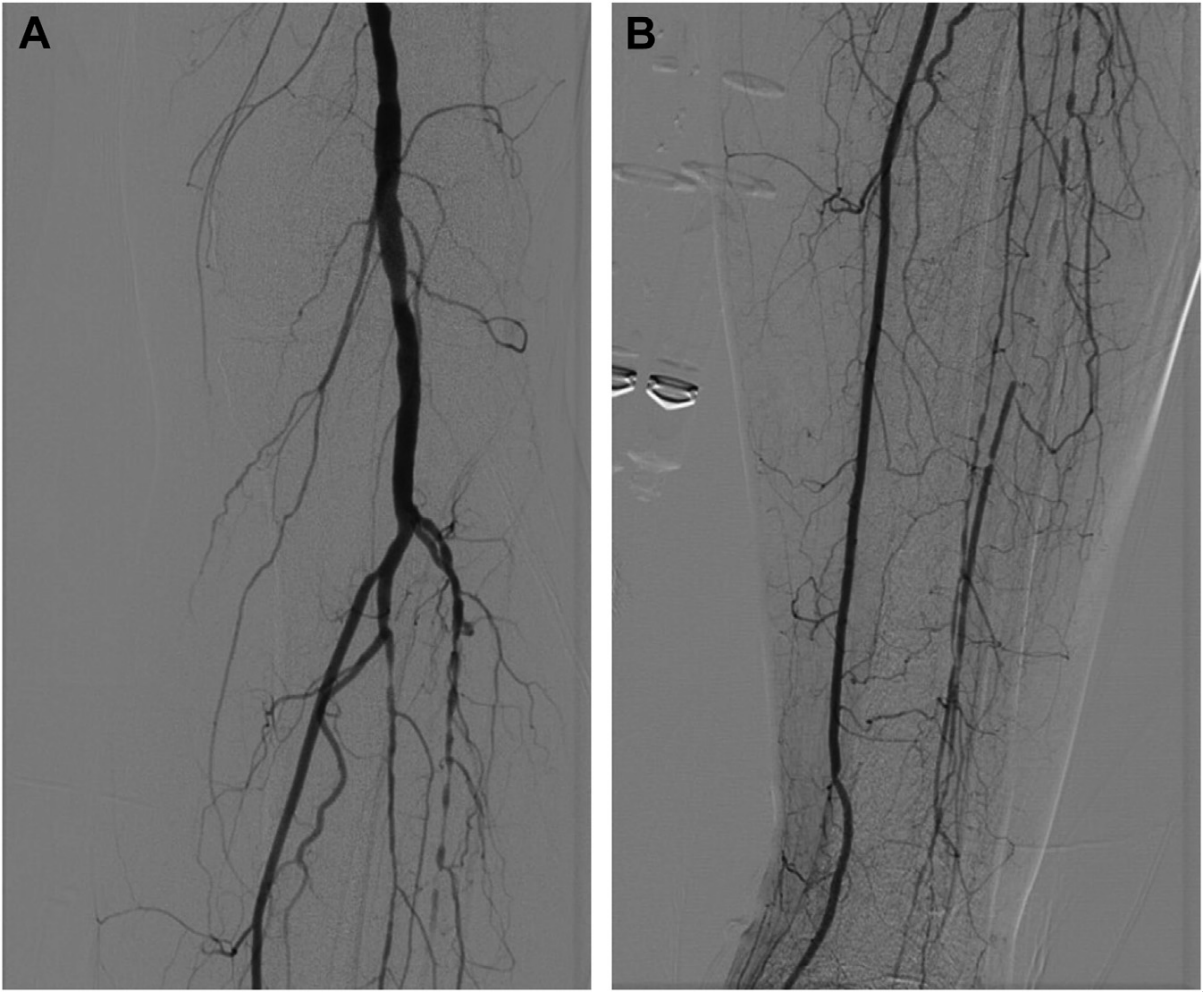
Angiographic evaluation of the affected limb revealed significant disease in the anterior tibial and peroneal arteries (**A**) with occlusion of both arteries below the knee (**B**).

A 69-year-old man with type 2 diabetes mellitus had initially presented to our multidisciplinary wound clinic with a wound on the plantar surface of his left heel/midfoot. The patient had previously been diagnosed with diabetic arthropathy and deformity of the left foot that was associated with stable collapse at the midfoot and loss of peripheral sensation. Angiographic assessment of the left limb demonstrated occlusion of the anterior tibial and peroneal arteries with single-vessel runoff to the ankle via the posterior tibial artery (Fig 1). Hemodynamic assessment at the time of angiography showed an ankle brachial index of 1.22 and a toe brachial index of 0.49 for the symptomatic, ulcerated limb. He subsequently underwent plastic reconstructive surgery to close the wound. However, his wound had recurred several times during the next 5 years, and he continued to receive treatment in the wound clinic. Although the patient understood the treatment options and the risk of limb loss through his prior experiences with wound debridement and procedures, he deferred further vascular treatment because he believed the wound did not significantly affect his quality of life. In addition to a notable vascular and wound history, the patient had diagnoses of hypertension, hyperlipidemia, and stage 2 chronic kidney disease (CKD).

At 5 years after angiography and foot reconstruction, the patient was enrolled in an imaging research study focused on evaluating the prognostic value of ^18^F-NaF PET/CT imaging to predict for calcific disease progression in patients with lower extremity PAD. The noninvasive hemodynamic assessment at enrollment revealed an ankle brachial index of 0.69 and a toe brachial index of 0.16 for the symptomatic, ulcerated limb. He was intravenously administered ^18^F-NaF (dose, 370 MBq) and underwent baseline ^18^F-NaF PET/CT imaging of the lower extremities 75 minutes later (Discovery 690; GE Healthcare, Chicago, IL). The patient underwent a follow-up calcium assessment of the left limb 1.5 years later using the same non–contrast-enhanced CT imaging protocol performed at baseline.

**Fig 2:**
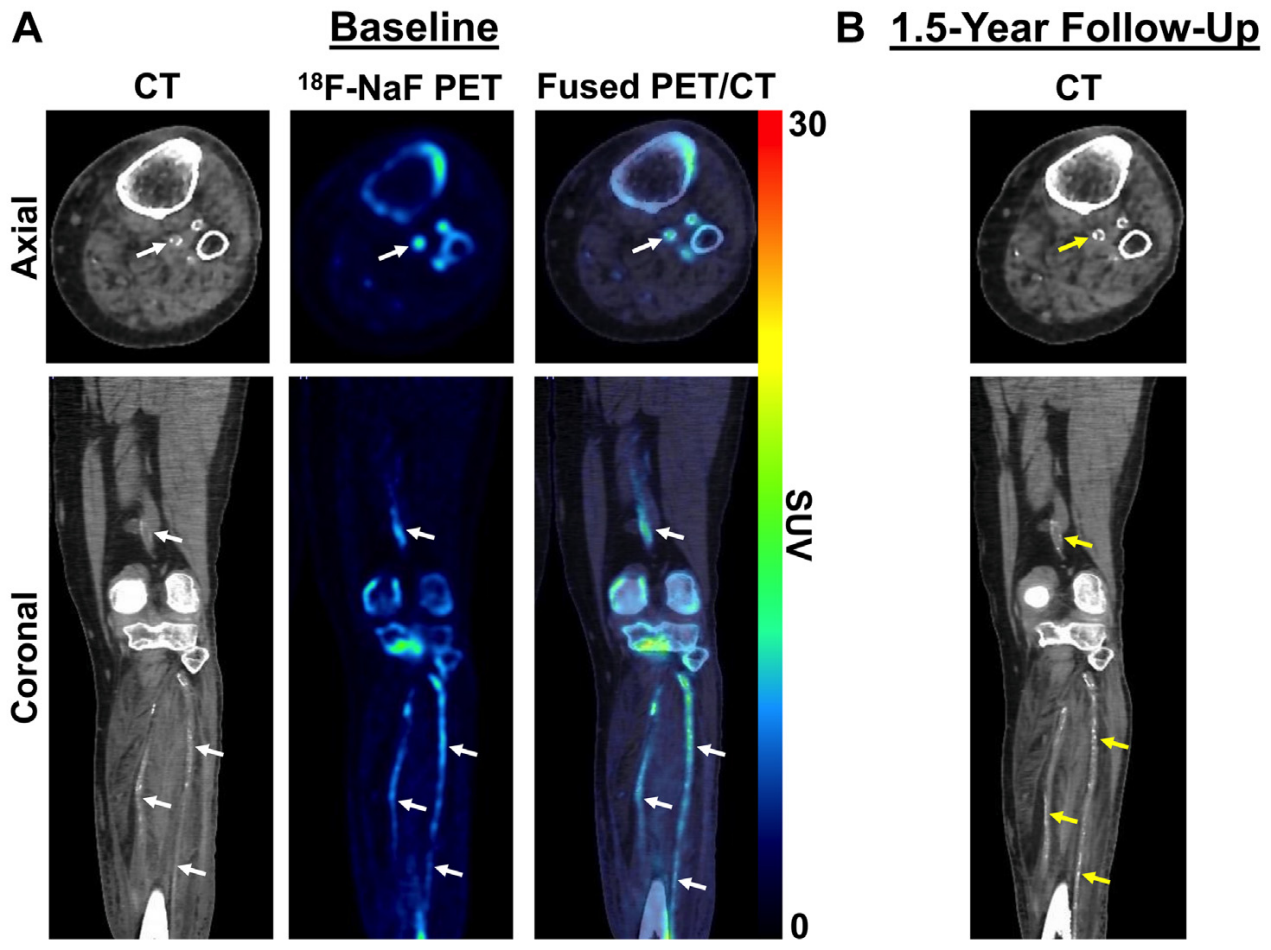
Fluorine-18 (^18^F)-sodium fluoride (NaF) positron emission tomography (PET)/computed tomography (CT) imaging of multivessel calcific disease progression. **A,** Baseline PET/CT imaging at study enrollment revealing various levels of CT-detectable calcium deposition in the popliteal, posterior tibial, anterior tibial, and peroneal arteries (*white arrows*) with active microcalcification occurring in multiple vessels, as shown by ^18^F-NaF PET imaging (*white arrows*). **B,** Follow-up CT imaging 1.5 years later demonstrating new calcium or progression of existing calcium within vascular regions (*yellow arrows*) that were previously identified as having elevated levels of ^18^F-NaF uptake (ie, active disease). *SUV,* Standardized uptake value.

**Fig 3:**
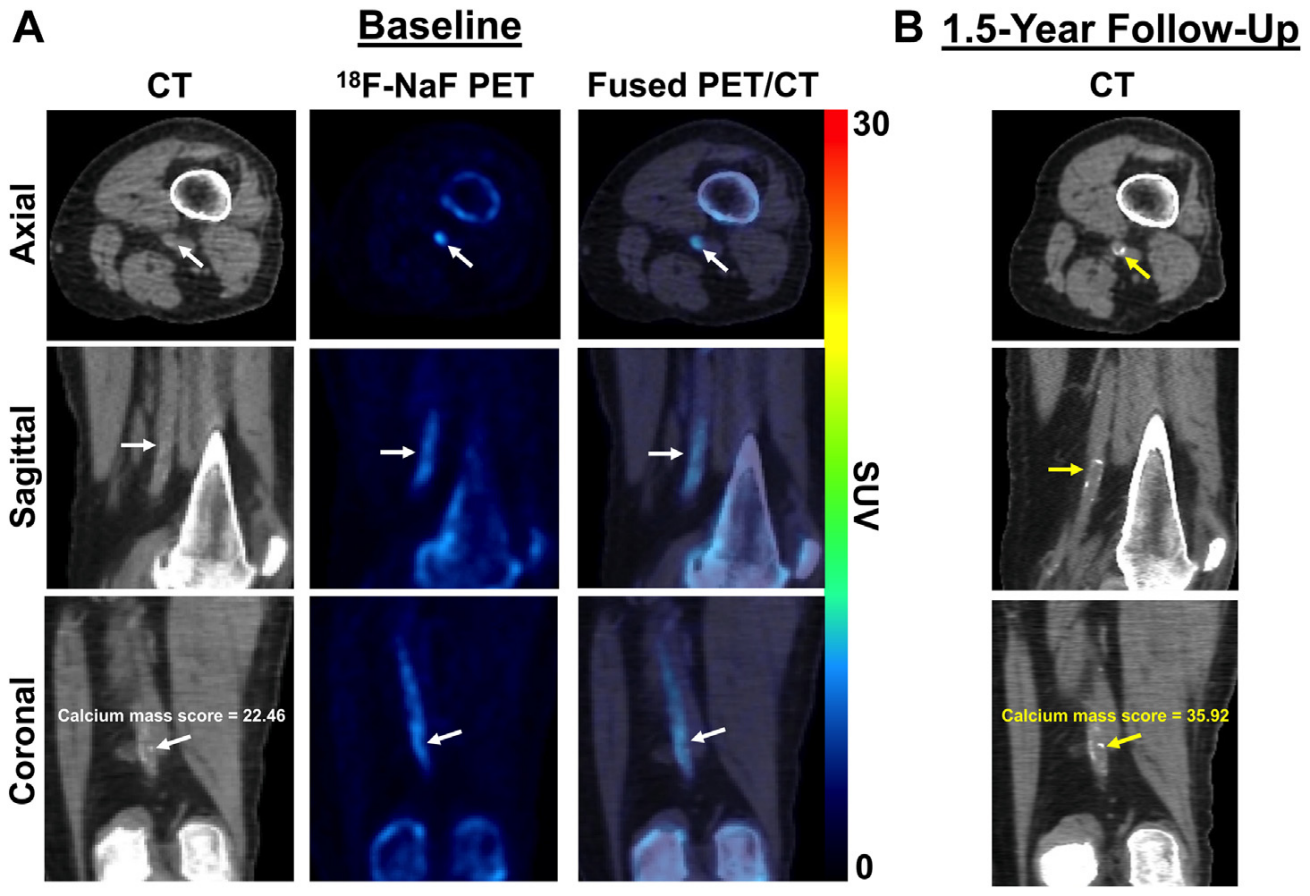
Fluorine-18 (^18^F)-sodium fluoride (NaF) positron emission tomography (PET)/computed tomography (CT) imaging of popliteal artery disease progression. **A,** CT imaging at study enrollment revealing minimal calcium deposition in the popliteal artery. In contrast, PET imaging demonstrated diffuse uptake of ^18^F-NaF, indicating an active process of popliteal artery microcalcification. **B,** Follow-up CT imaging demonstrated new calcium deposition in the popliteal artery where ^18^F-NaF PET imaging had revealed active microcalcification 1.5 years earlier. *SUV,* Standardized uptake value.

**Fig 4:**
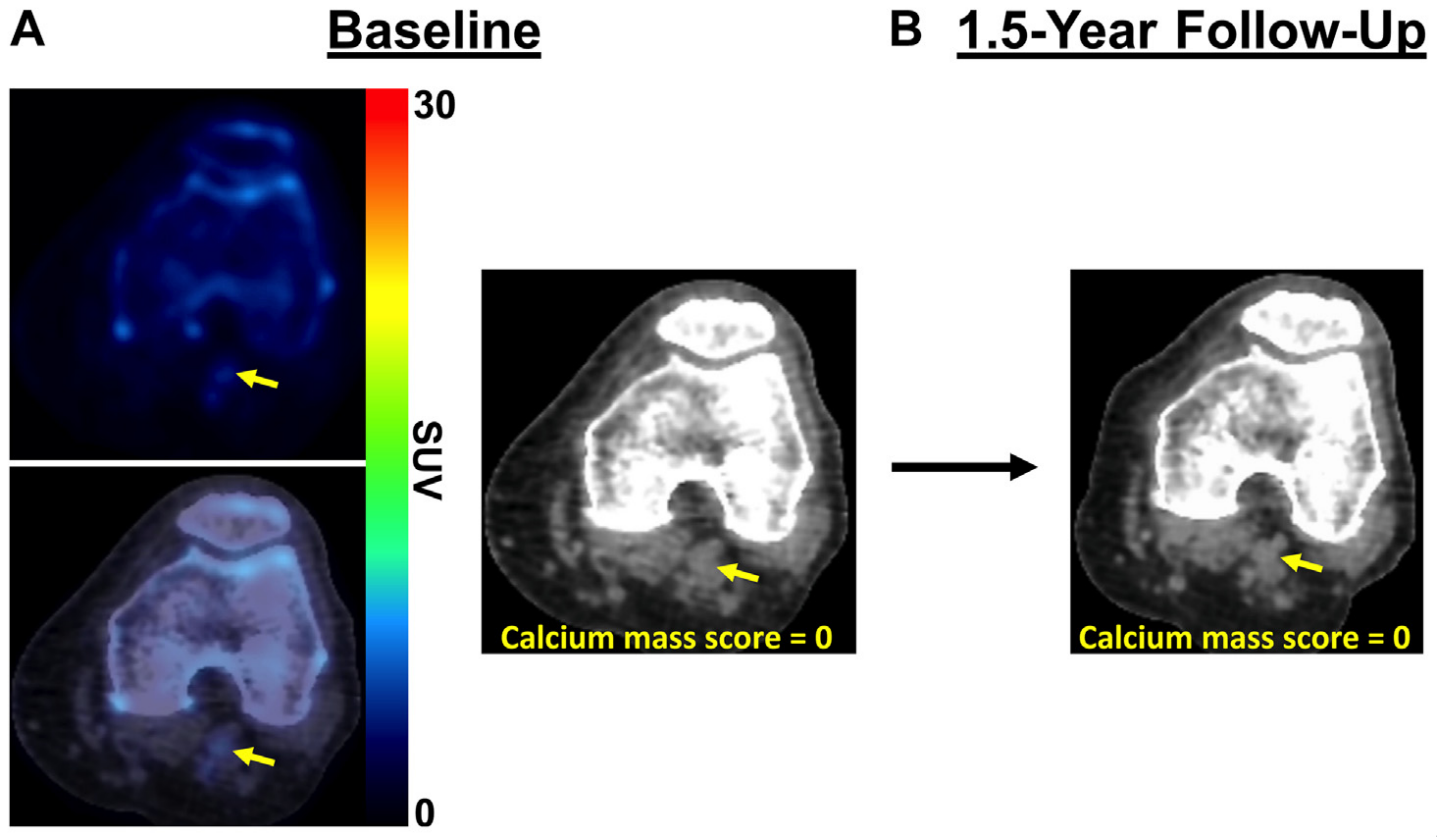
Positron emission tomography (PET)/computed tomography (CT) imaging revealing minimal uptake of fluorine-18 (^18^F)-sodium fluoride (NaF) in a region of the popliteal artery at baseline **(A)** that corresponded to no change in the calcium mass score **(B)** at 1.5-year follow-up. *SUV,* Standardized uptake value.

**Fig 5:**
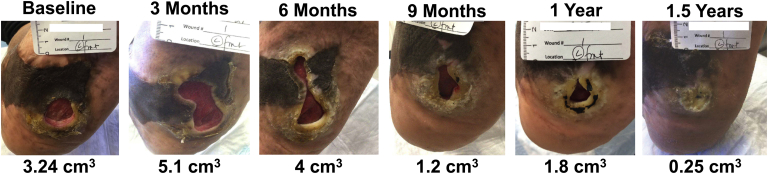
Serial photographs of the patient's chronic diabetic foot wound during the 1.5-year study timeline. Evidence of prior reconstructive surgery is apparent, with slow, but progressive, healing of the wound.

Baseline ^18^F-NaF PET/CT imaging revealed focal uptake of ^18^F-NaF (ie, active microcalcification) in multiple arterial sites of the left limb (Fig 2, *A*). The corresponding follow-up CT images acquired 1.5 years later demonstrated increases in vascular calcification within the same arterial sites previously identified as having increased ^18^F-NaF uptake (Fig 2, *B*). The popliteal artery was specifically identified as having minimal calcium with elevated ^18^F-NaF uptake at baseline (Fig 3, *A*), which later aligned with the same region of the popliteal artery, which had significant calcium 1.5 years later (Fig 3, *B*). Further analysis of ^18^F-NaF uptake and calcium progression in a separate region of the popliteal artery showed minimal ^18^F-NaF uptake at baseline that corresponded with no subsequent progression of calcium 1.5 years later (Fig 4). In addition to the various levels of calcium progression in multiple lower extremity arteries, the patient had progressed to stage 4 CKD at the 1.5-year follow-up. The foot ulcer on the plantar surface of the left foot was stable and showed slow improvement during the duration of the 1.5-year study timeline (Fig 5).

## Discussion

The presence and severity of a lower extremity calcium burden has traditionally been evaluated with standard of care imaging modalities such as CT; however, calcium detected on CT images represents established calcific disease. Alternatively, ^18^F-NaF PET/CT imaging allows for noninvasive monitoring of active arterial microcalcification and provides complementary molecular insight into the earlier stages of calcific disease progression.^6^ However, this technique has only recently been applied to the lower extremities,^7^, ^8^, ^9^ with only one study specifically focusing on evaluating patients with PAD.^9^ In a recent case, Eisert et al^10^ applied ^18^F-NaF PET/CT imaging for a patient with PAD and suggested that uptake of ^18^F-NaF might be elevated during the remodeling of lower extremity calcific aneurysms and in below-the-knee arteries. However, to the best of our knowledge, no study has assessed the prognostic value of ^18^F-NaF PET-detected active microcalcification for predicting subsequent CT-detectable calcific disease progression in multiple lower extremity arteries or evaluated calcium progression in patients with PAD. Thus, in the present report, we have built on the existing literature by describing the case of a 69-year-old man with CLTI who had undergone ^18^F-NaF PET/CT imaging for baseline evaluation of active vascular microcalcification and follow-up evaluation of calcium progression 1.5 years later. Our observation that calcific disease progression had occurred in multiple lower extremity arteries previously identified as having elevated uptake of ^18^F-NaF also builds on recently reported studies that have demonstrated ^18^F-NaF PET/CT imaging can predict for calcium progression in the coronary arteries,^11^ femoral arteries,^8^ and aorta.^12^ Additionally, the present patient's calcific disease progression, which occurred in sequence with his CKD progression, supports a recent study that demonstrated the contributions of CKD to active vascular microcalcification (ie, ^18^F-NaF uptake) in the lower extremities of patients with PAD.^9^

Angiographic evaluation of the patient's left limb years earlier had revealed single-vessel runoff via the posterior tibial artery. This prior information, combined with delayed, yet progressive, healing of his left foot wound, might suggest that the patient's posterior tibial artery had remained patent enough to support limb and tissue viability. However, as the findings from the present report have shown, the posterior tibial artery demonstrated elevated uptake of ^18^F-NaF at baseline and showed calcific disease progression on CT imaging 1.5 years later. Thus, this molecular imaging strategy might possess value for identifying at-risk vessels that could be the last remaining patent vessel for preserving limb integrity, which would be especially critical for patients with CLTI such as in the present report. Additionally, increasing the clinical application of ^18^F-NaF PET/CT imaging to PAD might increase future opportunities for personalized medicine for patients deemed at high risk of progressing to symptomatic PAD (claudication) or CLTI because of preexisting cardiovascular risk factors, offer a noninvasive biomarker for testing responses to novel PAD therapies, and facilitate our understanding of the specific contributions of traditional PAD risk factors, such as diabetes and CKD, to calcific progression in those with PAD.

## Conclusions

Hybrid PET/CT imaging with ^18^F-NaF detected early evidence of active microcalcification in multiple vessels within the symptomatic, ulcerated limb of a patient with CLTI that resulted in the appearance of new, or progression of, CT-detectable arterial calcium 1.5 years later. Our findings suggest that ^18^F-NaF PET/CT imaging can be used to predict calcific disease progression on a vessel-by-vessel or lesion-by-lesion basis in patients with CLTI, thus providing a noninvasive imaging strategy for assessing the risk of lower extremity disease progression and potentially enabling future improvements to personalized medicine for patients with PAD.
